# Celebrating 10 years of #RSCPoster

**DOI:** 10.1039/d5sc90028j

**Published:** 2025-01-31

**Authors:** Natalie Cotterell, Patrick A. J. M. de Jongh, Timothy Noël, Tanja Junkers, C. Malla Reddy, Athina Anastasaki, Edward Randviir

**Affiliations:** a Royal Society of Chemistry Thomas Graham House (290) Science Park, Milton Road Cambridge CB4 0WF UK rscposter@rsc.org; b Flow Chemistry Group, van’t Hoff Institute for Molecular Sciences (HIMS), Universiteit van Amsterdam (UvA) 1098 XH Amsterdam The Netherlands; c Polymer Reaction Design Group School of Chemistry, Monash University 17 Rainforest Walk Clayton VIC 3800 Australia; d Department of Chemistry, Indian Institute of Technology Hyderabad Kandi Sangareddy Telangana 502284 India; e Laboratory of Polymeric Materials Department of Materials ETH Zurich HCI G523 8093 Zurich Switzerland; f Department of Natural Sciences, Dalton Building Chester Street Manchester M1 5GD UK

## Abstract

#RSCPoster is an annual, 24 hour poster conference held online each March. Formerly on Twitter (now X), #RSCPoster saw the move to LinkedIn in 2024 and saw record levels of participation and engagement from communities around the world. The #RSCPoster 2025 edition celebrates 10 years since the inauguration of this popular, global poster conference. Here, we look back at the history of the event and growing engagement over the years, showcase some of the fantastic content we have seen from the events and encourage you to get involved in our next event.

## Abstract 2

Here, we look back at the history of #RSCPoster and growing engagement over the years, showcase some of the fantastic content we have seen from the events and encourage you to get involved in our next event.

## Graphical abstract

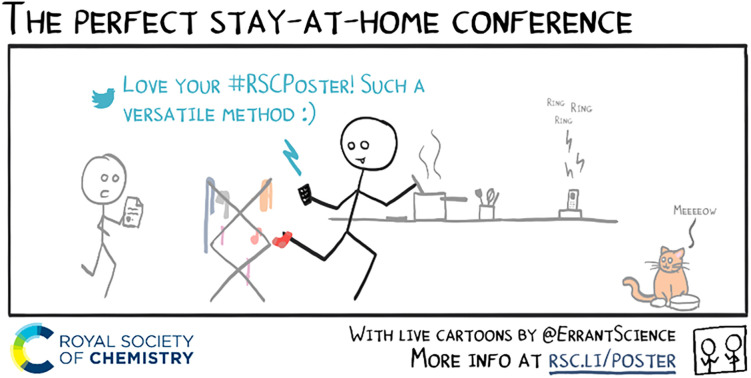

## The beginning: #RSCAnalyticalPoster

#RSCPoster originated from the success of the early concept #RSCAnalyticalPoster, developed through collaborations between the Royal Society of Chemistry (RSC), Dr Matthew Baker (University of Strathclyde, UK), Professor Craig Banks, Dr Edward Randviir and Dr Sam Illingworth (Manchester Metropolitan University, UK) in 2015.

The #RSCAnalyticalPoster event was hosted on Twitter (now X), with participants instructed to upload images of a poster to their Twitter profile with the hashtag #RSCAnalyticalPoster. As such, interested people could filter all tweets with the hashtag and view all content under this banner. The discussions were “open” for 24 hours from 9 am GMT on 5th February 2015. In this time period, anybody could reply to user contributions through direct replies to the poster upload, which opened multiple dialogues between contributors and interested people. Using open source Twitter analytics from followthehashtag.com, the first event saw over 80 posters uploaded to Twitter, from at least 9 separate countries, contributing over 1700 individual Tweets over the event period. The organizers considered the format to allow cross-continental discussion, and helped researchers communicate their ideas quickly and succinctly. This was one of the first online-only conferences, and the vision of this event was to bring together participants from all over the world to share their latest work in the field of analytical chemistry. The event was intended to provide an experimental space for researchers on a free-of-charge basis, hopefully inviting early-career researchers especially to contribute new ideas to the scientific discourse.

## #RSCPoster through the years

After initial success, a repeat of the event came in 2016, then in 2017 it was broadened in scope to incorporate the many other disciplines within chemistry, from the traditional physical, organic, and inorganic, through to more modern areas such as environmental and nanotechnology. This also necessitated a change in the name to #RSCPoster, and the addition of subject-specific hashtags (*e.g.* #RSCEng) for ease of filtering tweets by discipline. As the event attracted more attention and grew bigger, the organizing committee grew, bespoke judging panels were arranged for each discipline (at least three for each category, hence 30 people judging posters by 2018!), sponsorship was sought to award prizes in each subject area, and a scientific committee were appointed to ensure that conversations were initiated for all contributions to minimize Twitter’s in-built biases, and to ensure exchanges were appropriate. By 2020, the event was being promoted as green and inclusive (Ref: https://pubs.rsc.org/en/content/articlelanding/2020/cc/d0cc90441d), and had expanded to 800 participants creating 32 million impressions. Twitter posters were evolving away from traditionally structured posters to more engaging designs that were suitably formatted for mobile devices and video content, as well as highlighting community-based issues within the chemical sciences (*e.g.*, mental health). Post-event participant surveys from the 2020 event demonstrated that cost was a factor in their own participation, as well as relevance to their interests, and the fact it was a free online event was attractive for many. In 2021, the #RSCLive concept was born, which involved hosting some live webinars during the 24 hour period to focus on community issues, such as “choosing your supervisor”, “pathways to professor” and “mental health in the chemical sciences”. The webinars were staggered throughout the 24 h period, at strategic points across the timeframe to maximize and maintain engagement of the wider event. For example, data showed that each year the initial “buzz” at the start of the event was where most activity took place, with significant drop off in contributions after 5 hours. Hence, the sessions were targeted to straddle the 5 hour time mark to maintain interest in the wider event.

In 2021, we also introduced #RSCPosterPitch for the first time to allow participants to not only provide a written summary of their posters but have the opportunity to pitch their poster in a fun and creative format. These videos were only short, around 60 seconds long, but were a way to communicate the research and their findings in a more engaging way. In addition to the subject category awards, the most popular #RSCPosterPitch received an award as selected for by the General Committee. We have loved seeing all the different interpretations of #RSCPosterPitch, incorporating graphics, songs, cartoons and props, and encourage participants over the next years of #RSCPoster to consider posting an #RSCPosterPitch.

#RSCPoster today is a flagship event for the RSC, maintaining the very essence of the original concept, through sharing of excellent posters within the chemical science community during a 24 hour period while remaining completely free of charge. From sharing solely analytical chemistry content, #RSCPoster now incorporates 14 categories spanning the chemical sciences, which align to the portfolio of journals offered by the RSC. Fig. 1 displays the number of registrants each year for the #RSCPoster event (or #RSCAnalyticalPoster in 2015 and 2016). The graph shows a significant acceleration in registered participant numbers for each #RSCPoster event, particularly after the expansion of #RSCAnalyticalPoster to #RSCPoster in 2017. The 2017 event saw 80 registrants and this has grown to over 1700 registrants for our 2024 event.

**Fig. 1:**
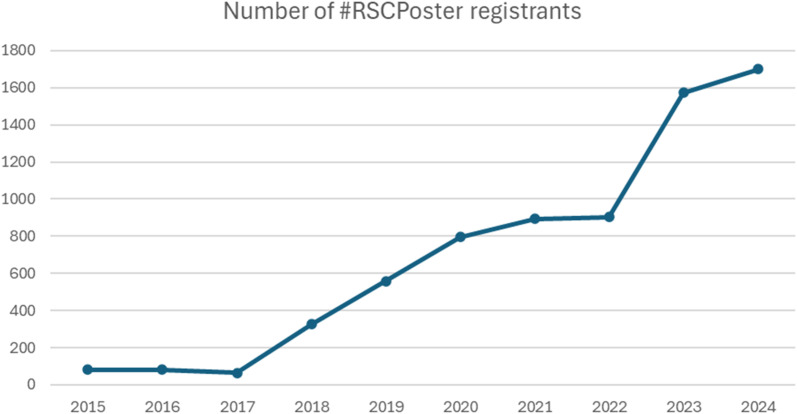
The number of #RSCPoster registrants has increased year on year. In 2017, the event was rebranded to #RSCPoster and in 2024, the event was moved from Twitter (now X) to LinkedIn.

## #RSCPoster from a diversity and inclusion perspective

One of the major advantages of holding a virtual scientific event is that, providing an internet connection is available, it is accessible for all participants equally. The 24 h duration of the #RSCPoster conference allows researchers from around the globe to participate, and subject chairs are selected with geographical diversity, so that at any given time, interaction is guaranteed. Moreover, since registration is free, no financial hurdles exist and this also brings a diversity of participants on other levels, such as those who may not otherwise have the funds for attending in-person conferences. Researchers from classically underrepresented groups can access information, enter discussions and present themselves to the communities, an opportunity they otherwise might not as easily have. This can be manifested in access from countries that typically do not have the funds available to travel to conferences, or to researchers who usually face significant hurdles in acquiring visas. Additionally, it is a possibility for researchers who might need to deal with caring responsibilities or a disability to put their scientific work into the spotlight equally. Last, but not least, the online format benefits people who may be neurodivergent or generally may not strive in crowded, in-person settings. All in all, the online #RSCPoster conference has a huge equalizing effect, which leads in large to a democratization of science and access to the discussion of new results. This can be quite empowering for members of minority groups. We encourage participants to embrace this diversity and to make connections that would not be made in more conventional settings.

**Fig. 2:**
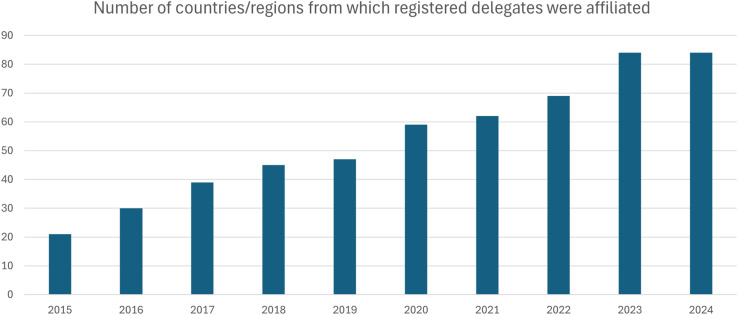
An increase in the number of countries/regions from which registered participants are based is seen year-on-year, with participants from 84 countries in 6 continents taking part in our 2023 and 2024 events.

Year-on-year, there are more registrants from more countries and regions across the world for the #RSCPoster event. In the last 5 years particularly, the global reach of #RSCPoster has grown significantly, with double the number of countries participating from 6 continents (see Fig. 2). This supports the values of #RSCPoster to bring together the global chemistry community to network with colleagues across the world.

**Fig. 3:**
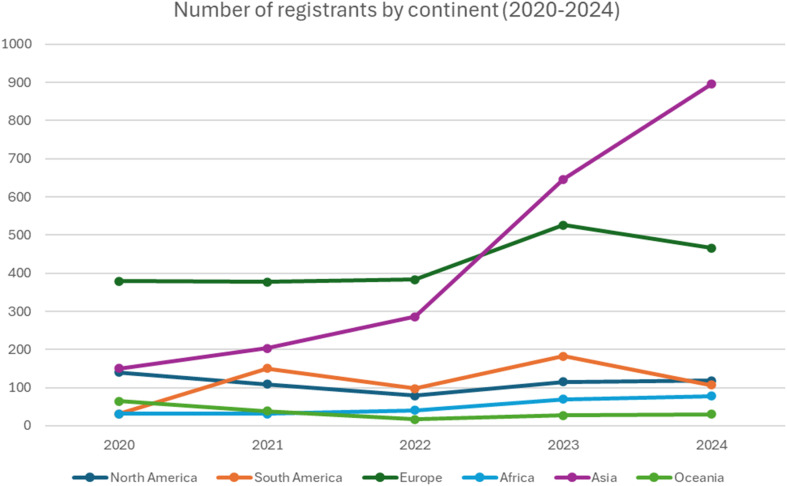
Number of participants per continent registered for #RSCPoster since 2020.

Fig. 3 summarizes the number of registrants by continent, based on their provided country and affiliation at registration, since 2020. Most notably in the last 5 years, participation from Asia has significantly increased and this is mainly due to the large increase in registrations from India. In 2020, there were 74 registrants from India and this has grown immensely to over 620 in 2024.

Overall, there has been an increase in participation from 6 continents since 2020 and, as alluded to above, delegates from more countries and regions globally have been involved in the events. In the last two years, participants have joined from countries and regions such as Eritrea, Cambodia, Guatemala, Fiji, Peru, Rwanda, Senegal, Yemen and Tanzania, supporting the event’s global reach. The RSC hope that the next decade of #RSCPoster brings participants from even more countries and regions together.

## #RSCPoster2024 and the move to LinkedIn

The 2024 edition of #RSCPoster took place from 4–5 March 2024 and saw the move of this popular and established Twitter (now X) poster conference to LinkedIn. After numerous years of being successful on Twitter (now X), there was the risk that participation levels may decrease, especially as a result of changes to Twitter (now X) policies. Despite this, there was a record breaking number of delegates registered for the 2024 event, with over 1700 delegates from 84 countries spanning 6 continents, compared to our 2023 event, which saw 1574 registrants from 84 countries (Fig. 1 and 2).

With the move to LinkedIn came a few changes, such as the launch of the RSC’s 17 LinkedIn showcase pages. These span the 14 #RSCPoster categories, with the 2024 addition #RSCFood, and general journal pages ChemComm, ChemSci and Sustainability.

To begin the 2024 #RSCPoster event, external organizers Tim Noël and Tanja Junkers chaired webinars on artificial intelligence in publishing and the use of pre-prints in the chemical sciences, respectively. The webinars received some excellent engagement with over 150 live viewers and 906 post-event viewers for the webinar on pre-prints and over 200 viewers and around 1500 post-event viewers for the webinar of the use of AI. In the webinar on preprints, the audience heard the perspectives of the editor of ChemRxiv, Ben Mudrak, RSC Executive Editor May Copsey and Prof. Robert Luxenhofer. The panel gave insights into preprint servers and their utility from an inside view, the view of journal editors and from the viewpoint of academic users. Many questions were raised from the community on the use of preprints and their changing role in academic publishing and a useful discussion evolved on the advantages of early publishing of scientific results, and how preprints can increase the quality of peer-reviewed articles. The webinar on artificial intelligence provided some interesting discussions from RSC Publishing Ethics team, Anna Pendlebury, and Nessa Carson (Astrazeneca), which is especially timely given the increase in use of AI, such as ChatGPT, in research. The panel discussed boundaries for using AI in research and publications, which gave some great insight into the broad use to this day. Given the change in platform, these webinars could be more easily viewed by participants and other members of the community live and post-event with an accessible recording on the RSC LinkedIn account without pre-event registration. The webinars also continued in the essence of discussing important topics and community issues that researchers may face in academia. We always welcome suggestions for new webinar topics for the coming years and encourage our community to contact the #RSCPoster team if there are any webinar topics they would like to see.

## Collaborations with ErrantScience

**Fig. 4:**
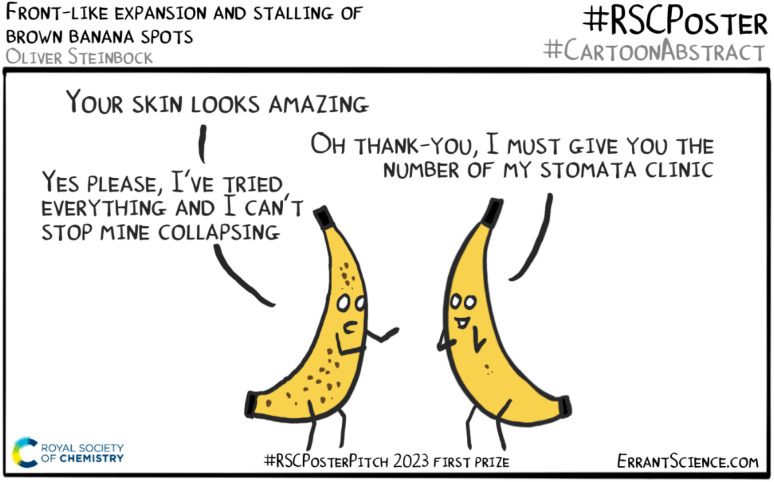
A cartoon celebrating the winning 2023 #RSCPosterPitch from Oliver Steinbock.

ErrantScience is a daily web cartoon blending humour and insight into the everyday life of researchers and scientists. With cartoons, they try to capture the ups and downs of lab work and academia, and explain the ever-complex world of science. Alongside this, ErrantScience also produces cartoons that help demystify and explain complex scientific ideas through live conference cartooning, outreach support, and making cartoon abstracts. Since 2015, Matt Partridge from ErrantScience has been providing this style of fun cartoons to #RSCPoster with yearly live cartoon sessions during the event (Fig. 4). This has changed over the years, but he tends to spend each #RSCPoster event reading furiously and drawing as many cartoons as he can.

Each year, Matt aims to produce somewhere between 20 and 30 ErrantScience cartoons in the 24 hours that #RSCPoster runs. These are typical single-panel cartoons that help to explain or highlight particularly interesting poster submissions to #RSCPoster. Some years, this has included extras such as videos, animated GIFs, collages, live streams, and time lapses. But the cartoons have always been the core part of ErrantScience’s work, and at the last count, there are now a little over 200 #RSCPoster cartoons.

We asked Matt what he enjoyed most about collaborating with #RSCPoster each year. *‘I think what I’ve enjoyed most about the event isn’t a single thing but two. One right from the start and one that came later. It’s going to sound like a cop-out but the thing I initially liked the most from #RSCPoster is the posters. When I started cartooning #RSCPoster, I was a participant with a poster submission of my own, and as I sat at my desk reading people’s questions and looking through the other posters, I started doodling cartoons. But over time I think the part I have enjoyed the most is the impact my cartoons have had both on #RSCPoster and research beyond. Each year there are more and more posters which include their own cartoons or are even wholly cartooned. And each year we get more and more messages from people showing us the cartoons they’ve started including in their posters, presentations, and even papers. I really believe science has space for creativity and silliness, and it’s so good to see a growing number of people we’ve inspired. I’m always happy to find work that I can’t cartoon because the researcher has already done a better job themselves!’*.

**Fig. 5:**
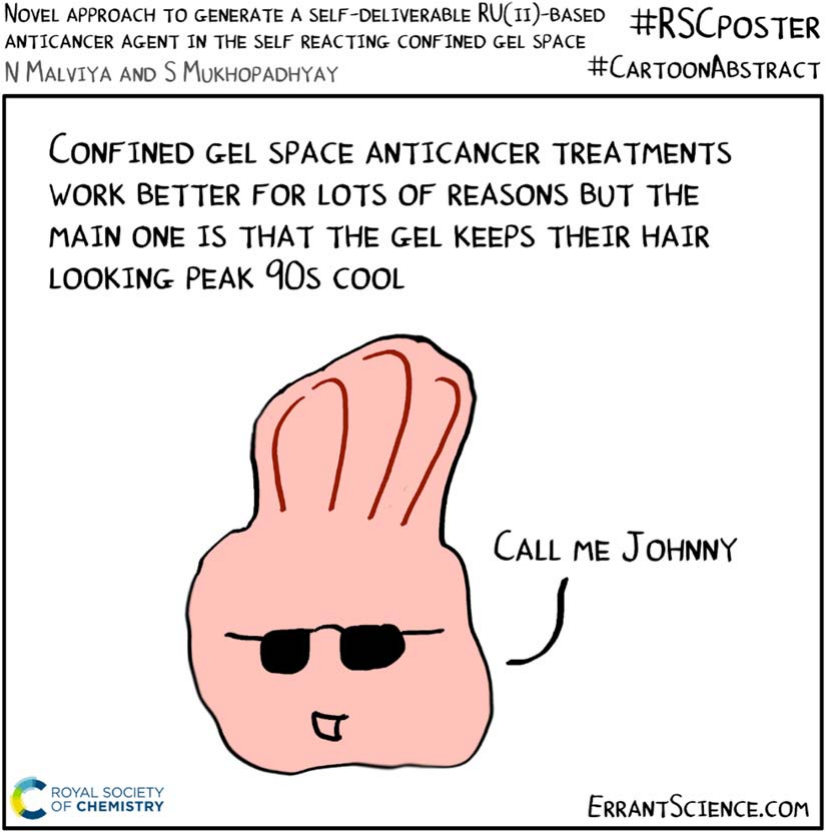
There are many sources of inspiration for #RSCPoster cartoons, including tv series.

**Fig. 6:**
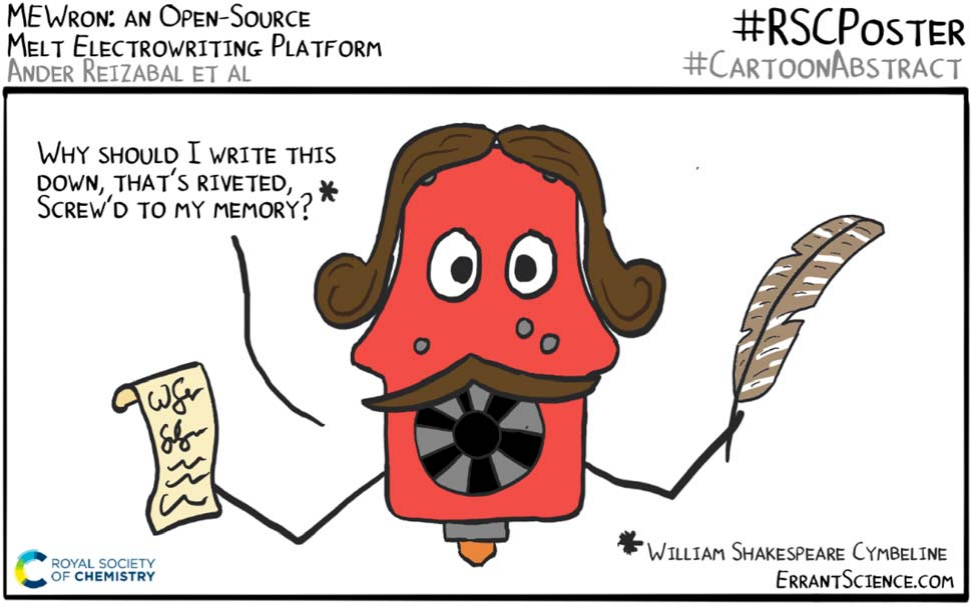
Some cartoons are inspired by historical figures.

**Fig. 7:**
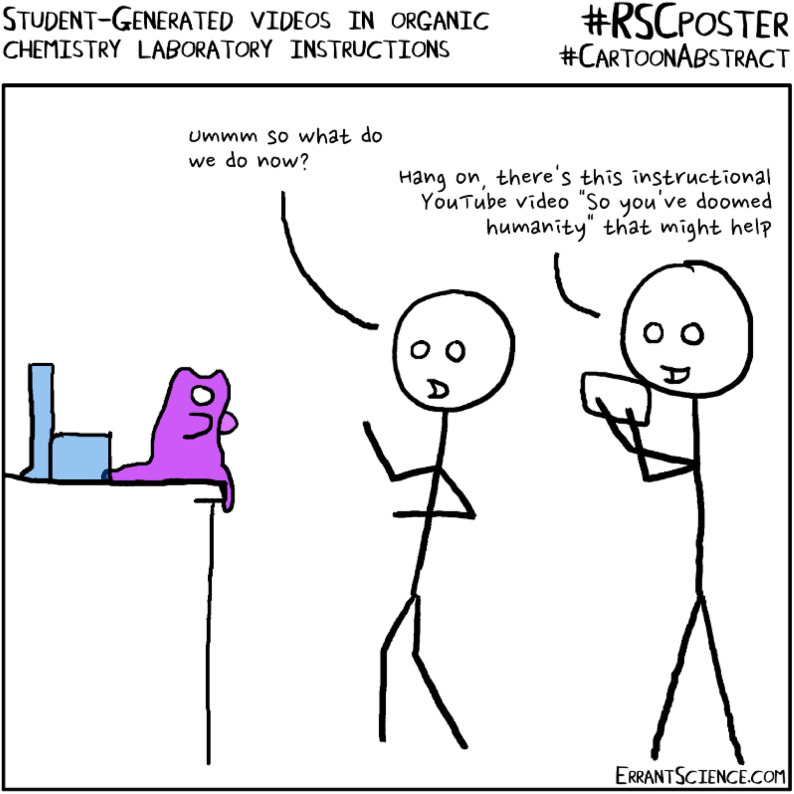
ErrantScience’s cartoons often show a playful take on what can happen in a lab.

**Fig. 8:**
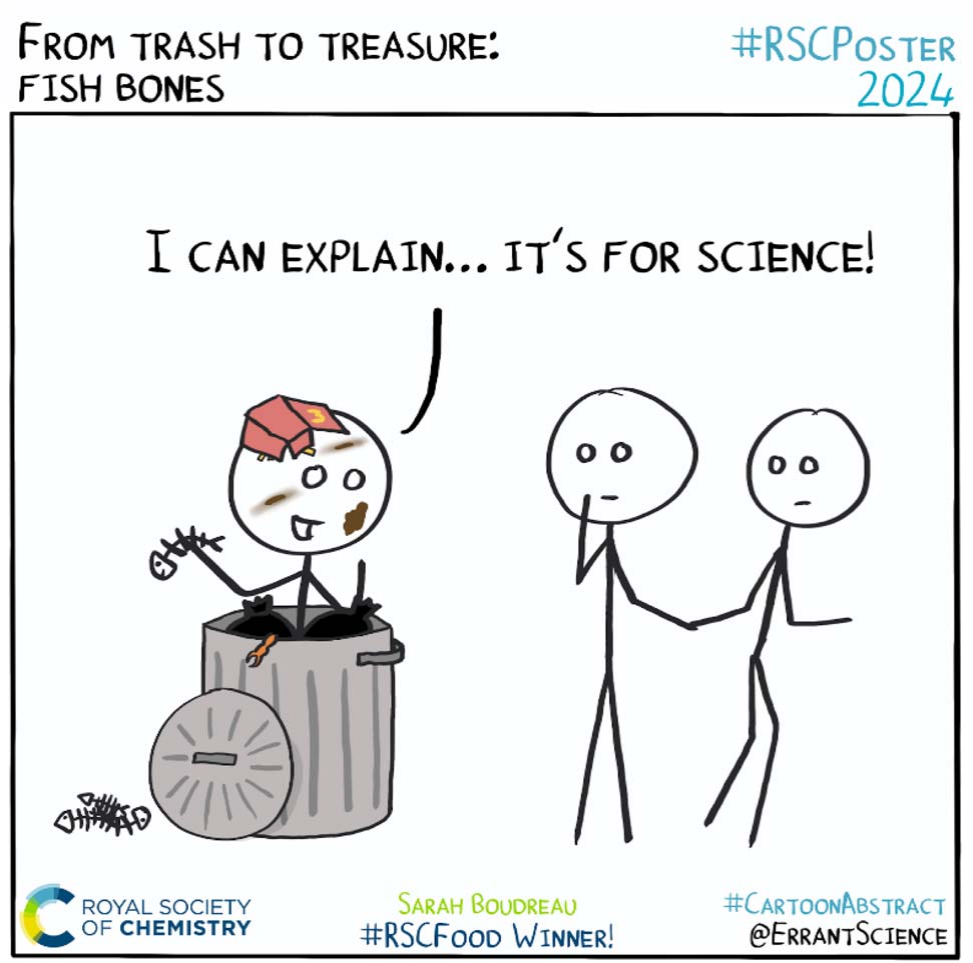
A cartoon to celebrate the 2024 #RSCFood winner Sarah Boudreau.

If you have not seen any of Matt’s ErrantScience sketches, we would encourage you to check them out (Fig. 5–8). They cover a wide range of themes and the level of creativity and wittiness is always impressive. We asked Matt where he finds the inspiration for all of these sketches. *‘I get inspired by the most random of places. I’ve drawn cartoons based on Lord of the Rings just because I happened to watch it the weekend before; others because of a small throwaway comment in a paper that stuck in my head. One poster was inspired by stepping on some Lego on the way back to my desk. My kids have also inspired more than one cartoon by saying “I bet you can’t draw X in a cartoon today”. I find challenges to be inspiring, so the idea is the more fun I have trying to find a cartoon that would work. Top tip for anyone wanting their poster cartooned: I respond well to “I bet you can’t do this one”’*.

*‘With over 200 cartoons made for RSCPoster, it’s very hard to choose a favourite. Looking back through the archive as the anniversary approaches, I remember things that inspired those cartoons and the posters they came from. Some were instant “oh wow, I know exactly what I want to do!!” Some ended up being an hour’s research, drafting, and editing, but all of them have been great. The animated ones I did in 2021/2022 were also so much fun’*.

## An honorary mention to our community

It is worth mentioning that the #RSCPoster events could not be as successful as they are without the support from our wonderful community. Each year, our teams of Subject Chairs get involved in the event by raising its visibility, encouraging registrants, engaging with posters and spending the time judging and deciding which posters should be awarded a prize post-event. We value the time they have given up supporting the smooth running of this flagship event. We also appreciate the help from our General Committee who promote the event and engage with participants during the 24 hour period. Finally, we would like to recognise and thank all past participants from the last 10 years who have presented a poster, attended the webinars, shared an #RSCPosterPitch and engaged with posters from other participants. The involvement and enthusiasm of our community is certainly what really makes #RSCPoster one of our favourite events of the year.

## Get involved in #RSCPoster

Never before been involved in an #RSCPoster event? Now is your chance! #RSCPoster is a chance to share your research from the comfort of your own home. No dress code, no travelling, no registration fees. We asked our current and previous external organisers and previous winners to share some top tips for preparing an #RSCPoster and for getting involved in the event. As #RSCPoster is an online conference with a broad audience, you have the chance to make your poster extra eye-catching by including graphics and cartoons, animations and splashes of colour. Check out our 10 top tips below to help you get the most out of the #RSCPoster event:

**(1) Keep it simple**

#RSCPoster attracts a broad audience and it is therefore worth making sure the science is understandable by non-experts in the area. Consider communicating science through graphics and figures rather than lots of text and focus on the main aspects of the work.

**(2) Make your poster stand out**

**Fig. 9:**
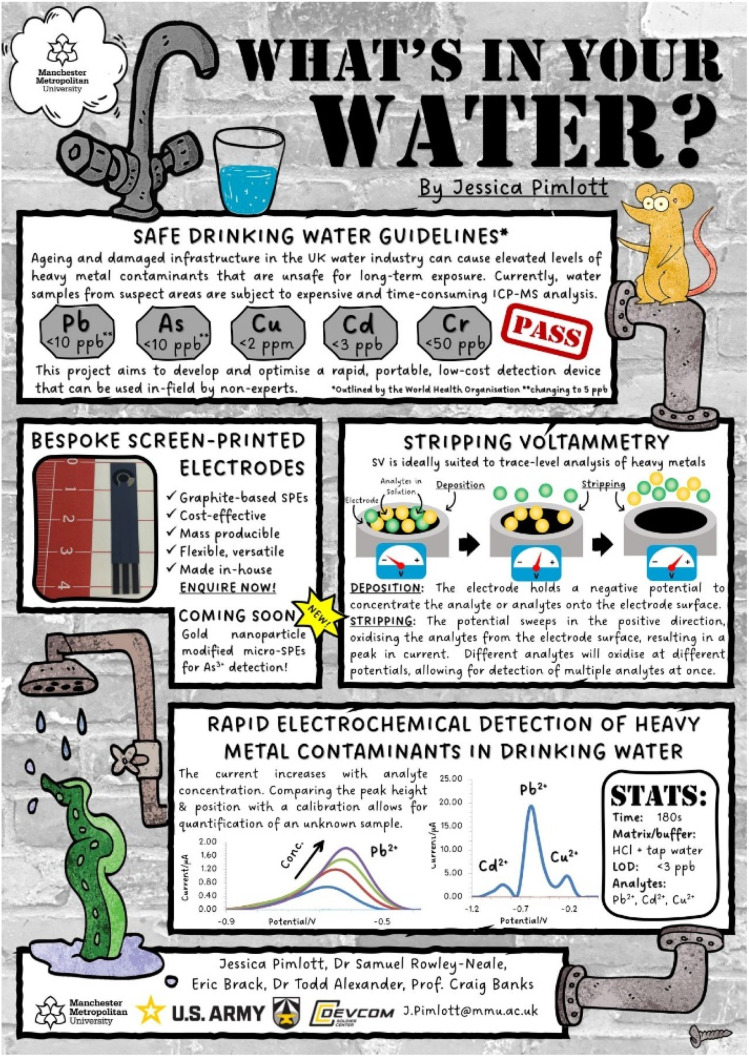
An example of an eye-catching poster by 2024 #RSCEnv winner Jessica Pimlott, reproduced with permission from the authors.

**Fig. 10:**
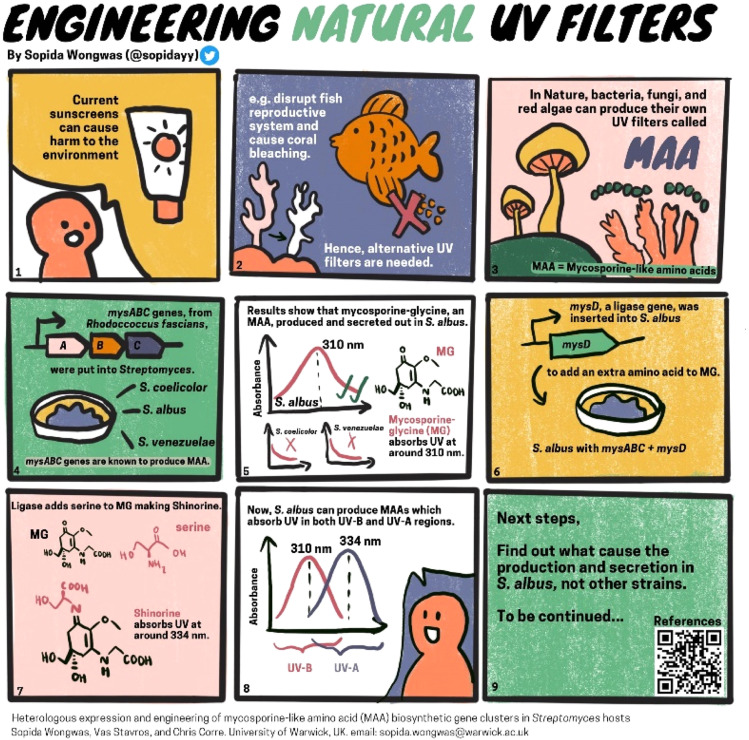
2023 #RSCChemBio runner-up Sopida Wongwas presented researchin the form of a comic strip, reproduced with permission from the authors.

During the 24 hour event, there are so many posters flooding our feeds, so make sure your poster is eye-catching (Fig. 9). Previous delegates have even made their posters into cartoon strips or infographics (Fig. 10), so there is plenty of room for creativity.

**(3) Why not post an #RSCPosterPitch?**

This is a short 1–2 minute video highlighting the importance of the research presented in the poster. Creativity is of course encouraged and we welcome songs, animations and props. There is also the opportunity to win a prize for the best #RSCPosterPitch.

**(4) Be attentive to font size and format**

As #RSCPoster is on a digital platform, the size of your poster will be a lot smaller than at an in-person conference. When preparing your #RSCPoster, make sure that the resolution of the images is high enough to be easily viewed on LinkedIn, and allows quality zoomability if required, and increase the size of your font and figures to be easily readable. Having less text and focusing on communication through figures gives you more room to go large.

**(5) Familiarise yourself with LinkedIn**

As #RSCPoster is being held on LinkedIn, it is worth checking out the platform beforehand and creating your account if you don’t already have one before the day. Follow the RSC LinkedIn account to make sure you don’t miss out on the webinars during the event, any updates and winner announcements. Follow the subject pages that you are interested in (*e.g.*, #RSCMat for materials-based research or #RSCFood for food-based research) so you can see and engage with other posters during the event.

**(6) Decide your subject area**

When posting your #RSCPoster, you will need to tag the official #RSCPoster hashtag along with the subject page for which you would like to be considered a prize. Not only this, but tagging our subject pages allows your work to be more discoverable by other researchers and participants working in the area who may want to network and ask questions about your work.

**(7) Register for free**

We encourage all delegates to register before the event to make sure their posters will be considered for a poster prize. Our committee of subject chairs will be scouring LinkedIn during the event, checking out posters, asking questions and ultimately judging the winners of the competition. Delegates are asked to select a subject category in which they wish to be considered for a prize, so you will need to select this at registration.

**(8) Get involved during the event**

The concept of #RSCPoster is to network, share research and engage in scientific debate, so don’t be shy and engage with other posters and delegates during the event. Ask questions, give feedback and learn about research that may not even be in your field. Join our free webinars given by external organisers during the event to learn about important topics related to academia.

**(9) Spread the word**

Help us to spread the word of our 2025 event by getting your friends, colleagues, group, societies, interest groups, *etc.*, involved. We want to make #RSCPoster as fun and inclusive as possible, so get involved, encourage others to get involved and let’s have our best year yet!

**(10) Find out more**

Check out our webpage and FAQs for more information.

## Hear it from our previous winners

So why get involved? #RSCPoster is a unique experience to engage with scientists across different fields, learn about research happening in other fields, and share your research without limitations of an in-person conference, and ultimately it is truly a fun event. But don’t just hear it from us, hear it from some of our previous #RSCPoster winners.

*#RSCPoster provided the opportunity to be creative and go beyond the traditional way of making posters for conferences and I prepared my poster in GIF format or video format instead of a traditional conference poster.*

**Vijay Kumar Jayswal, Canada, 2021 #RSCMat runner-up**

*What I loved most was how the event encouraged creativity. I tried to make the most of the social media platform for presenting research. In 2022, I put some animated elements into my poster, whilst in 2023, I opted for a comic-style presentation. This event presents an excellent opportunity to enhance your science communication skills for a diverse audience.*

**Sopida Wongwas, UK, 2023 #RSCChemBio runner-up**

*I truly think this event is for everyone, but it is especially great for ECRs as it allows exposure and connection to a wide audience without the need for travel funds or visas.*

**Silke Asche, UK, 2023 #RSCDigital winner**

*What I enjoyed most was the vibrant sense of community and the opportunity to connect with fellow researchers from diverse backgrounds. The lasting connections I made significantly enriched my research journey.*

**Chandra Shekhar, India, 2024 #RSCInorganic runner-up**

*My RSC posters have been used as teaching tools as far as UNSW, and I began delivering science communication workshops facilitated by connections made *via* the competition.*

**Kelly Brown, UK, 2021 #RSCAnalytical winner**

*I’m always looking forward to this event, to learn about studies I had no idea about, to nurture my own research with novel ideas, and to discuss my own ideas with brilliant minds.*

**Silvana Silva-Aguirre, Mexico, 2022 #RSCDigital winner**

*It challenges you to think creatively about how to simply communicate your research—not just to scientists, but also to other curious social media users.*

**Paul Kimani, Kenya, 2021 #RSCEnv runner-up**

*#RSCPoster is a fantastic platform for fostering curiosity and making chemistry and science accessible to everyone—something I deeply believe in.*

**Anaïs Pitto-Barry, France, 2020 #RSCEdu winner**

## Outlook for #RSCPoster

It is evident that the early concept of #RSCPoster has grown into an immense success and has paved the way for digital conferencing. The #RSCPoster organisers are always looking for ways to adapt to the ever-changing social media landscape to ensure that our reach is still globally significant and that our events are as accessible as they can be. The decision to move from Twitter (now X) to LinkedIn in 2024 after establishing the competition there since 2015 came with challenges and risks, but the support of the community, organisers and committees meant that this paid off and we are excited to once again hold #RSCPoster on LinkedIn in 2025. Our aims are to encourage more participants from more countries and regions across the world to get involved in sharing their research and so far we have seen significant increases in engagement year-on-year over the last decade. We hope to see a record number of participants sharing their latest research in a creative way, whether through a stand-out #RSCPoster or through an innovative and engaging #RSCPosterPitch in our 2025 event. Thank you to everyone who has been involved in any of our #RSCPoster events over the last 10 years and we look forward to seeing how #RSCPoster develops over the next decade!

